# Effect of Ulinastatin Combined with Xingnaojing Injection on Severe Traumatic Craniocerebral Injury and Its Influence on Oxidative Stress Response and Inflammatory Response

**DOI:** 10.1155/2022/2621732

**Published:** 2022-01-10

**Authors:** Zexin An, Yong Yin, Lei Zhang, Bo Wang, Tao Cui, Meng Li, Jianwei Zhuo, Jing Zhang, Kai Wang, Wenwen Zhang, Meng Ji, Jilin Sun, Yinong Xu

**Affiliations:** ^1^Department of Neurology, Cang Zhou Central Hospital, Cangzhou, Hebei Province, China; ^2^Department of Magnetic resonance imaging, Cang Zhou Central Hospital, Cangzhou, Hebei Province, China; ^3^Department of Orthopedics, Cang Zhou Central Hospital, Cangzhou, Hebei Province, China; ^4^Department of Neurosurgery, the Fourth People's Hospital of Taizhou, Jiangsu Province, China

## Abstract

**Objective:**

This study is aimed at exploring the effect of ulinastatin combined with Xingnaojing injection on severe traumatic craniocerebral injury and its influence on oxidative stress response and inflammatory response in patients.

**Methods:**

A total of 100 patients with severe traumatic craniocerebral injury admitted to our hospital from January 2018 to January 2020 were selected and equally assigned into a study group (50 cases) and a control group (50 cases) according to a random sampling method. Patients in study group received treatment of ulinastatin combined with Xingnaojing injection, while those in control group were treated with ulinastatin only. The study compared the two groups on the oxidative stress response, inflammatory response, the therapeutic effect, and the incidence rate of adverse reactions.

**Results:**

It is observed that patients in study group obtained lower levels of free cortisol (FC) and norepinephrine (NE) in the serum and higher level of total thyroxine (TT4) after treatment compared with those in control group with significant difference (*P* < 0.05); in the meantime, they were examined to have significantly fewer oxidative stress response products, lower serum inflammatory factor level, and serum indicator levels of craniocerebral injury as opposed to those in control group, suggesting significant differences (*P* < 0.05); study group demonstrated higher treatment response rate and lower incidence rate of adverse reactions compared with control group with a significant difference (*P* < 0.05).

**Conclusion:**

The study found that ulinastatin combined with Xingnaojing infection has a significant effect in the treatment of severe traumatic craniocerebral injury, which can reduce the degree of craniocerebral injury and the level of inflammatory factors in the serum of patients. It is worthy of being promoted and applied clinically.

## 1. Introduction

Severe traumatic craniocerebral injury is a common critical and severe disease, which usually results from a direct or indirect external blow or jolt to the head or body, leaving craniocerebral tissue and blood vessels being seriously injured and morphology changes such as skull fracture [1, 2]. It causes stress reaction and trauma in patients and produces imbalance of cerebral oxygen metabolism and disturbance of internal environment. The incidence of severe traumatic craniocerebral injury is critical with high disability and mortality rate, and complications can easily occur during the treatment [3, 4]. Patients with severe traumatic craniocerebral injury will have cerebral hypoxia-ischemia, cellular inflammatory response, and other conditions. Cellular inflammatory factors will accelerate nerve cell death, destroy cranial nerve function, and prevent neuronal regeneration, resulting in central nervous system damage [5, 6]. Studies have shown that ulinastatin combined with Xingnaojing injection has a good therapeutic effect on severe traumatic craniocerebral injury and can reduce the injury of patients. In order to further study the effect of ulinastatin combined with Xingnaojing injection in the treatment of severe traumatic craniocerebral injury and its influence on oxidative stress response and inflammatory response, 100 patients with severe traumatic craniocerebral injury admitted to our hospital from January 2018 to January 2020 were selected as the study subjects. Research data are shown below.

## 2. Materials and Methods

### 2.1. General Information

A total of 100 patients with severe traumatic craniocerebral injury admitted to our hospital from January 2018 to January 2020 were selected as the study subjects and equally assigned into a study group (50 cases) and a control group (50 cases) according to a random sampling method. The patients in control group were at the age range of 51~72 years old while patients in study group were 63 to 77 years of age. There was no significant difference in the general data between the two groups (*P* > 0.05) (as shown in Table 1).

#### 2.1.1. Inclusion Criteria

Patients' traumatic craniocerebral injury happened within 24 hours; patients with severe traumatic craniocerebral injury had been diagnosed by brain computerized tomography (CT) examination; this study was approved by the hospital ethics committee (No. 2017-231-16); all patients and their families were informed of the purpose and method of the study and had signed the informed consent form.

#### 2.1.2. Exclusion Criteria

Exclusion criteria are as follows: patients with contraindications to drugs; those with tumors in the brain; those with epilepsy; those with severe liver and kidney dysfunction; and those with major infectious diseases.

### 2.2. Methods

Patients in the two groups had conventional treatment and examination mainly including reducing the intracranial pressure and maintaining the airway patency. Meanwhile, they were given oxygen inhalation and hemostasis to maintain the acid-base balance and electrolyte balance and necessary nutritional support. Their blood glucose and blood pressure were monitored and controlled at all times [7, 8]. If patients require immediate surgery, decompressive surgery or craniotomy for evacuating hematoma should be performed in a timely manner.

Besides conventional treatment, patients in control group took ulinastatin injection (SFDA Approval No.: H19990133 Manufacturer: Guangdong Tianpu Biochemical Pharmaceutical Co., Ltd.; Specifications: 50,000 units/vial). Patients was administrated with 2∗10 5 U of ulinastatin injection, and 100 mL normal saline for intravenous drip, once every 8 hours.

Patients in study group were treated with ulinastatin combined with Xingnaojing injection on the basis of conventional treatment, and the usage amount and method of ulinastatin were consistent with that of control group. It included 30 mL of Xingnaojing (SFDA Approval No.: Z53021639; Manufacturer: Dali Pharmaceutical Co., Ltd.; Specifications: 10 mL) and 100 mL of normal saline for intravenous drip, once a day [9, 10].

### 2.3. Observation of Indicators

The levels of free cortisol (FC), norepinephrine (NE), and total thyroxine (TT4) were examined by chemiluminescence kit; the levels of interleukin-1 (IL-1), interleukin-6 (IL-6), and tumor necrosis factor-*α* (TNF-*α*) were measured by immunoradiometric assay; the levels of neuron-specific enolase (NSE), myelin basic protein (MBP), and S100 proteins were measured by enzyme-linked immunosorbent assay (ELISA); and the levels of Malondialdehyde (MDA), 8-isoprostane (8-iso-PGF 2*α*), and Heat Shock Protein 27 (HSP27) were measured by radio-immunoprecipitation kit. Then, we compared the two groups on the therapeutic effect and the incidence of adverse reactions.

### 2.4. Statistics

The statistical analysis was conducted with SPSS 20.0 software, and Graphics drawing was carried out with GraphPad Prism 7 (GraphPad Software, San Diego, USA). Measurement data were represented as ($\bar{x}\pm s$) and analyzed by *t*-test. Enumeration data were represented as (*n*, %) and analyzed by *χ*^2^ test. *P* < 0.05 indicated a statistically significant difference.

## 3. Results

**Figure 1:**
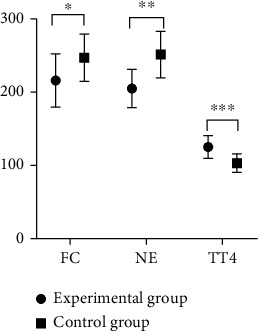
Comparison of the levels of stress response hormone in the serum. Note: the abscissa represents the stress response hormone in the serum; the ordinate indicates the amount. The study group had (216.1 ± 36.2) mg/L of FC, (205.2 ± 26.1) mg/L NE, and (125.3 ± 15.5) mg/L TT4; the control group had (247.2 ± 32.4) mg/L of FC, (251.5 ± 31.6) mg/L of NE, and (103.2 ± 12.7) mg/L of TT4. ^∗^ indicated that there was a significant difference in FC between the two groups; *T* = 4.527; *P* < 0.001. ^∗∗^ indicated that there was a significant difference in the NE between the two groups; *t* = 7.988; *P* < 0.001. ^∗∗∗^ indicated that there was a significant difference in TT4 between the two groups of patients; *t* = 7.799; *P* < 0.001.

**Figure 2:**
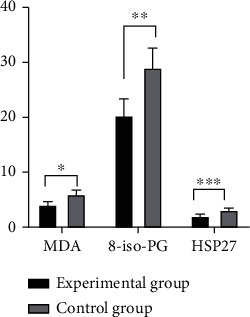
Comparison of oxidative stress response products. Note: the abscissa represents the oxidative stress response products, and the ordinate represents the amount. The study group had (4.07 ± 0.61) mg/L of MDA, (20.35 ± 3.01) mg/L of 8-iso-PG, and (2.04 ± 0.34) mg/L of HSP27; the control group had (6.01 ± 0.77) mg/L of MDA, (29.02 ± 3.58) mg/L of 8-iso-PG, and (3.13 ± 0.36) mg/L of HSP27. ^∗^ indicated that there was a significant difference in MDA amount between the two groups of patients, *t* = 4.527; *P* < 0.001. ^∗∗^ indicated that the 8-iso-PG amount was significantly different between the two groups of patients, *t* = 13.107; *P* < 0.001. ^∗∗∗^ indicated that HSP27 amount was significantly different between the two groups of patients, i.e., *T* = 15.565; *P* < 0.001.

**Figure 3:**
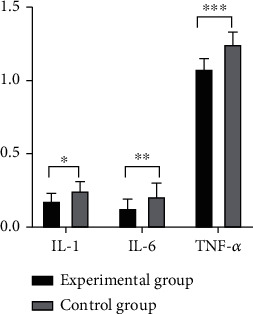
Comparison of the levels of serum inflammatory factors. Note: the abscissa represents the serum inflammatory factor indicators; the ordinate represents the levels. Patients in study group displayed levels of serum factor IL-1, IL-6, and TNF-a as, respectively (0.18 ± 0.05), (0.13 ± 0.06), and (1.08 ± 0.07); those in control group had levels of serum factors IL-1 and IL-6 being (0.25 ± 0.06), (0.21 ± 0.09), and (1.25 ± 0.08), respectively. ^∗^ indicated that the levels of serum factor IL-1 between the two groups of patients were significantly different, *T* = 6.337; *P* < 0.001. ^∗∗^ stated that there was a significant difference in the level of serum factor IL-6 between the two groups of patients, *t* = 5.229; *P* < 0.001. ^∗∗∗^ indicated that the levels of serum factor TNF-a of the two groups of patients were significantly different, *t* = 11.308; *P* < 0.001.

**Figure 4:**
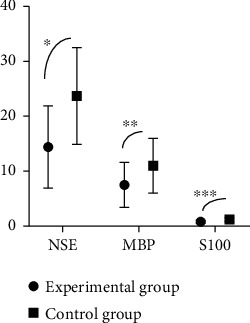
Comparison of serum indicators of craniocerebral injury. Note: the abscissa indicates the serum indicators of craniocerebral injury; the ordinate indicates the levels. Patients in study group had the levels of serum indicators NSE, MBP, and S100 being, respectively (14.4 ± 7.5), (7.5 ± 4.1), and (0.8 ± 0.3) after the treatment; those in control group had the levels of NSE, MBP, and S100 being, respectively (23.7 ± 8.8), (11.0 ± 5.0), and (1.2 ± 0.3) after the treatment. ^∗^ indicated that there was a significant difference in NSE level between the two groups, *t* = 5.687; *P* < 0.001. ^∗∗^ indicated that a significant difference was observed in MBP levels between the two groups, *t* = 3.827; *P* < 0.001. ^∗∗∗^ indicated that two groups had significantly different S100 levels, *t* = 6.667; *P* < 0.001.

**Figure 5:**
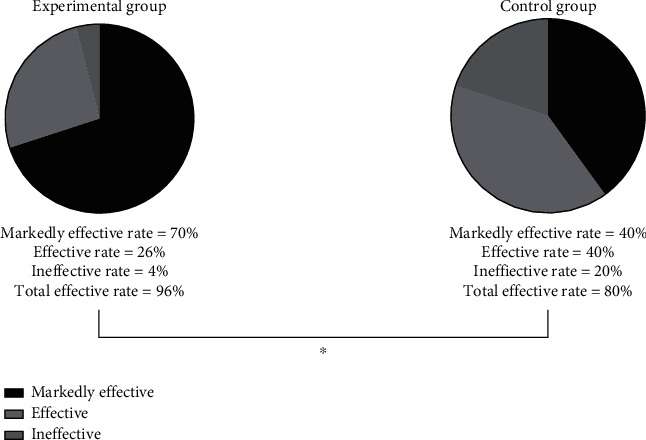
Comparison of treatment response rate. Note: the study observed 35 significantly effective cases, 13 effective cases, and 2 ineffective cases, respectively, in study group, while there were 20 significantly effective cases, 20 effective cases, and 10 ineffective cases, respectively, in control group. ^∗^ stated that the overall response rate of 96% in study group was greater than that of 80% in control group, and the difference was statistically significant (*P* < 0.05).

1. After the treatment, patients in study group were observed to have lower levels of FC and NE in the serum and higher level of TT4 compared with those in control group, with significant difference (*P* < 0.001) (as shown in Figure 1).
2. The oxidative stress response products of patients in study group was significantly lower than that of patients in control group after the treatment, displaying a significant difference (*P* < 0.001) (as shown in Figure 2).
3. It was detected that the levels of serum inflammatory factors of patients in study group were all lower than those of patients in control group after the treatment, suggesting a significant difference (*P* < 0.001) (as shown in Figure 3).
4. Patients in study group manifested lower levels of serum indicators of craniocerebral injury in contrast to those in control group. The difference was statistically significant (*P* < 0.001) (as shown in Figure 4).
5. After the treatment, study group had a significantly higher treatment response rate as opposed to control group with a significant difference (*P* < 0.05) (as shown in Figure 5).
6. The incidence of adverse reactions in study group was lower than that in control group, displaying a significant difference (*P* < 0.05) (Table 2).

## 4. Discussion

Previous surveys and studies on major diseases show that the incidence of craniocerebral injury accounts for 0.1 percentage of the incidence of major diseases in China, 20% of which are severe traumatic craniocerebral injury, a common clinical emergency [11, 12]. If such patients have a long-time coma which cause great damage to their neurological function, a severe stress response may happen with many complications [13–15]. Severe traumatic craniocerebral injury develops rapidly, so timely treatment should be conducted to reduce patients' mortality and disability rate.

Ulinastatin is an endogenous protective substance reducing the release of elastase, trypsin, and some lipid hydrolases to inhibit the expression of inflammatory factors [16, 17]. Meanwhile, it can also prevent the generation of oxygen free radicals, improve the microcirculation in patients, and reduce the damage of tissues and organs under inflammatory and ischemic conditions so as to increase the tolerance of the liver to hypoxia [18–20]. Currently, ulinastatin has been widely used in the treatment of severe traumatic craniocerebral injury and severe pancreatitis for its good therapeutic effect and high safety [21, 22]. Xingnaojing is made from Angong Niuhuang Pills which has the effects of cooling blood and detoxicating, clearing away heat and toxic material and eliminating phlegm for resuscitation. Its injection can act directly on patients' central nervous system to regulate the energy metabolism, improve the blood flow and oxygen supply, and reduce craniocerebral tissue hematoma [23, 24]. Relevant studies have demonstrated that Xingnaojing can control the high fever response in patients with severe craniocerebral injury and help patients tide over the critical stage.

The study showed that patients in study group had lower levels of FC and NE in the serum and higher level of TT4 compared with those in control group, with a significant difference (*P* < 0.05); they also had fewer oxidative stress response products and lower serum inflammatory factor level in contrast to those in control group, and the differences were statistically significant (*P* < 0.05). It demonstrates that the treatment of ulinastatin combined with Xingnaojing injection can effectively inhibit the release of inflammatory factors so as to control the level of inflammatory factors. Patients' neurons will be damaged due to increasing serum levels after craniocerebral tissue being injured. In addition, we observed that the levels of serum indicators NSE, MBP, and S100 in study group were significantly lower than those in control group, and the differences were statistically significant (*P* < 0.05), indicating that the tested treatment can effectively reduce the craniocerebral tissue injury and accelerate the rehabilitation of patients. And the treatment response rate of patients in study group remained higher than that in control group, whilst the incidence of adverse reactions was lower than that of control group, suggesting significant differences (*P* < 0.05). This demonstrated that the therapeutic effect of ulinastatin combined with Xingnaojing was better than that of ulinastatin alone. BreeDara et al [25]. stated in their study that ulinastatin combined with Xingnaojing injection treatment of severe craniocerebral tissue injury could reduce the level of serum inflammatory factors in patients; results of which were similar to the conclusion of this study and fully demonstrated the scientific rationality of our findings.

Taken together, ulinastatin combined with Xingnaojing injection has a significant effect in the treatment of severe traumatic craniocerebral injury by reducing the degree of craniocerebral injury and lowering the level of inflammatory factors in the serum of patients. It is worthy of promotion and application.

## Figures and Tables

**Table 1:** General information ($\overset{¯}{X}\pm s$).

Group	Study group	Control group	*T*/*χ*^2^	*P*
Gender (male/female)	25/25	26/24	0.04	0.84
Age (years)	69.65 ± 4.31	70.22 ± 4.60	0.64	0.52
Height (cm)	165.09 ± 5.12	165.61 ± 5.73	0.48	0.63
Strike (case)	5	4	0.12	0.73
Car accident (case)	20	23	0.37	0.55
Drop (case)	20	19	0.04	0.84
Others (case)	5	4	0.12	0.73

**Table 2:** Comparison of incidence of adverse reactions (*n*, %).

Group	Number of people	Neurogenic edema	Stress ulcer	Blood creatinine increased	Total incidence
Study group	50	2% (1/50)	0 (0/50)	0 (0/50)	2% (1/50)
Control group	50	6% (3/50)	4% (2/50)	4% (2/50)	14% (7/50)
*χ* ^2^		1.041	2.04	2.04	4.891
*P*		0.307	0.153	0.153	0.027

## Data Availability

No data were used to support this study.
